# Where you live, what you do: depression differences among diverse Chinese nongmin through cognitive openness

**DOI:** 10.3389/fpsyt.2025.1433949

**Published:** 2025-01-31

**Authors:** Li He, Jiangyin Wang, Yang Yang, Zhilu Tian, Liu Jiang

**Affiliations:** ^1^ Zhongnan University of Economics and Law, School of Philosophy, Wuhan, Hubei, China; ^2^ Central China Normal University, School of Politics and International Studies, Wuhan, Hubei, China; ^3^ Wuhan University, School of Marxism, Wuhan, Hubei, China

**Keywords:** CFPS, subsistence farmers, agricultural laborers, rural non-agricultural workers, rural-to-urban migrant workers, depression

## Abstract

**Objective:**

This study aims to investigate the depression levels among Workers with Agricultural Hukou (WAH) in China, considering their varied living environments, types of work, and social discrimination experiences. It specifically addresses the research question: Is there a significant discrepancy in depression levels among different subgroups within WAH?

**Methods:**

The study utilizes data from the China Family Panel Studies (CFPS) for the years 2018 and 2020. To estimate the depression levels and their variances across different WAH subgroups, we employed three analytical methods: Ordinary Least Squares, Propensity Score Matching, and Two-Stage Least Squares.

**Results:**

Our findings indicate that all WAH subgroups experience higher levels of depression compared to Workers with Non-Agricultural Hukou (WNAH). Among the WAH subgroups, the depression levels, in ascending order, are observed in Rural-to-Urban Migrant Workers (RUMW), Rural Non-Agricultural Workers (RNAW), Subsistence Farmers, and Agricultural Laborers. Notably, these differences in depression levels may be influenced by the mechanism of cognitive openness.

**Conclusion:**

The study concludes that there are significant disparities in depression levels among WAH subgroups. Understanding these differences is crucial for targeted mental health interventions and for addressing the broader implications of social discrimination and work-related stress among agricultural workers in China.

## 1 Introduction

In China, there exists a large group referred to as “nongmin”. In English, terms such as “Farmer”, “Peasants”, and “Peasantry” are commonly used to denote “nongmin”, but these terms are often used interchangeably and fail to fully capture the essence of “nongmin” in the Chinese context. In China, “nongmin” possess not only occupational attributes but also identity attributes. To accurately investigate this group, it’s imperative to delineate its boundaries. Occupation, geographic location, and hukou serve as three effective indicators for this delineation (1), with the hukou system playing a pivotal role in classification. Therefore, this study uses hukou as the primary defining criterion. China’s hukou system, which originated in 1955 (2), differentiates between agricultural and non-agricultural hukou, establishing clear and institutionalized boundaries between rural and urban residents in China involving rights and responsibilities (3). Within this system, workers with agricultural hukou (WAH) are the focus of this research.

Yet, characterizing WAH merely by their agricultural hukou status oversimplifies the reality. The seventh national census by the National Bureau of Statistics of China revealed that while the hukou urbanization rate stood at 45.4% in 2020, the actual urbanization rate of the permanent population was approximately 63.89%. This discrepancy highlights that out of roughly 770 million individuals with agricultural hukou, only 510 million reside in rural locales, with a mere 170 million actively engaged in agriculture. This demonstrates significant differences in living locations and job types within the WAH population, necessitating further segmentation. Based on this reality, this study categorizes the WAH population into four specific subgroups: Subsistence Farmers and Agricultural Laborers, who are engaged in agricultural work and reside in rural areas, with Subsistence Farmers primarily relying on their land for livelihood, and Agricultural Laborers being employed by others; and Rural-to-Urban Migrant Workers (RUMW) and Rural Non-Agricultural Workers (RNAW), differentiated by their urban versus rural residencies.

Despite historically higher depression rates among WAH due to their lower social stature, economic limitations, and scarce medical resources compared to other social classes and occupational groups (4, 5), debates persist regarding WAH’s depression levels (6, 7). This controversy stems from the diversity of living and working environments of WAH in different countries and regions. This study focuses on the situation of WAH in China. Affected by the hukou system, only those with urban hukou in China have access to social welfare benefits including housing, education, medical care, and retirement (3), making WAH a vulnerable group. Notably, RUMW, despite residing in cities, are still deemed temporary urban inhabitants (8) with severely limited support in social welfare, labor rights, and health insurance benefits (9). This vulnerability is not only reflected in the acquisition of social resources but is also widely believed to exist at the psychological health level (10). As for the classification of WAH in this research: first, Subsistence Farmers, primarily found in developing countries and relying on small-scale agricultural production by family units, have been relatively understudied regarding their depression levels. Second, Agricultural Laborers, common in both developed and developing nations, have been more closely examined and are thought to experience elevated depression levels (11). Third, with China’s ongoing economic evolution and the subsequent diversification of the WAH, the mental health of RUMW and RNAW, particularly depression, has garnered increasing scholarly attention. Given that most WAH in non-agricultural roles only returns to rural settings upon aging (12), research on RNAW primarily targets mental health issues among the elderly (13), often linking them to heightened depression rates.

In conclusion, compared to Workers with Non-Agricultural Hukou (WNAH), WAH and their subgroups in China display higher depression rates, forming the empirical groundwork for this study. Building on this premise, the research further delves into the depression disparities among the WAH subgroups and the underlying causes of these potential variances.

## 2 Theoretical analysis and hypotheses

Ecosystem Mental Health Theory, as an emerging interdisciplinary approach, emphasizes the importance of considering mental health within a broad ecological context (14). This theory posits that an individual’s mental health status is influenced by various levels of their ecosystem, such as individual (15), family (16), society (17), and environment (18), and the interactions between these levels determine the support resources available to the individual and the pressures they face, thereby affecting their mental health condition.

Specifically, the significant differences in depression levels among the various WAH subgroups in China can be analyzed from both individual and environmental perspectives. Engaging in agricultural work often means lower work income (19) and susceptibility to negative impacts from sudden changes in the work environment (especially the natural environment) (20). Therefore, Subsistence Farmers and Agricultural Laborers might exhibit higher depression levels due to being in less favorable ecosystems. Although Subsistence Farmers and Agricultural Laborers face similar environmental challenges, the employed Agricultural Laborers, due to lower work autonomy, are more susceptible to physical and mental harm (21), thereby increasing the risk of depression. For WAH engaged in non-agricultural work, the differences in their depression levels stem from their different environments. Compared to RNAW, RUMW, by moving from rural areas to urban work, improve their employment opportunities and economic status, which may contribute to their well-being (22) and reduce their depression levels. This indicates that the differences in ecosystems in which the various WAH groups reside lead to certain differences in depression levels. Therefore, the following hypothesis is proposed:

Hypothesis 1: There are significant differences in depression levels among Subsistence Farmers, Agricultural Laborers, Rural-to-Urban Migrant Workers, and Rural Non-Agricultural Workers.

Further analysis reveals that the individual differences in work types and environmental differences in living locations exhibited by the four subgroups of WAH not only lead to variations in depression levels, but also indicate significant differences in the social culture, cognitive thought, and values they are exposed to. These differences can further explain the underlying causes of their depression disparities. Accordingly, this study will explore the internal mechanisms behind the depression level differences among the subgroups of WAH from the perspective of cognitive openness. In this study, cognitive openness is defined as the degree to which individuals accept new ideas, new experiences, and uncertainty (23). This concept reflects an individual’s cognitive and emotional responses when facing environmental changes or social and cultural differences (24). Among the subgroups of WAH, cognitive openness may influence their acceptance of traditional cultural values (25), which in turn may impact their mental health and depression levels to some extent. Relative to other societal groups, Chinese WAH typically attain lower educational levels (26) and are profoundly impacted by traditional Chinese cultural norms (27), such as traditional family ethics and the thought repression caused by adverse social culture, which significantly negatively affect their depression levels (28).

According to the Ecosystem Mental Health Theory, socio-cultural and values have a significant impact on an individual’s mental health status (29). Specifically, workers or residents in urban areas are more likely to accept modern education and ideologies, which enables them to have a higher capacity for critical thinking and re-evaluation of traditional cultural values, thereby typically exhibiting higher cognitive openness. These individuals’ higher cognitive openness may allow them to better adapt to changes brought about by modern society, reducing the occurrence of depressive symptoms (30). In contrast, WAH from remote rural areas, due to limited educational opportunities and the deep-rooted influence of traditional culture, generally exhibit lower cognitive openness, making them more susceptible to psychological distress and more difficult to adapt to life pressures (31). Therefore, cognitive openness may affect depression levels by altering an individual’s perception of social culture and traditional values. Based on this, we propose the following hypothesis:

Hypothesis 2: The degree of cognitive openness acts as the underlying mechanism influencing depression level differences among the WAH subgroups.

While prior research has explored the impact of traditional concepts and cognitive perceptions on the depression levels of the WAH, its scope has been confined to specific groups. Therefore, based on data from the China Family Panel Studies, this study not only assesses and compares the depression levels across WAH subgroups but also explores the mechanism of cognitive openness. Specifically, first, the baseline regression analysis confirmed the differences in depression levels among the WAH subgroups. Second, to ensure the robustness of the results, robustness checks were conducted, and propensity score matching (PSM) and instrumental variables methods were employed to address potential endogeneity issues, which verified the stability of the baseline regression results. Finally, a mediation model was used to examine the mediating role of cognitive openness in the depression differences among the WAH subgroups.

Moreover, the potential contributions of this study mainly include: theoretically, first, it verifies the applicability of the Ecosystem Mental Health Theory to the health issues of the Chinese WAH; second, further exploration and enrichment of the research on health issues of the WAH through discussing the differences in depression levels among the WAH subgroups; third, mechanism analysis from the perspective of cognitive openness, providing a new angle for exploring the internal logic behind the depression levels and differences among the WAH subgroups. Practically, it can provide references for public agencies to devise tailored welfare policies for WAH and their subgroups.

## 3 Data, variables and method

### 3.1 Data analysis

#### 3.1.1 Data management

The data for this investigation was derived from the China Family Panel Studies (CFPS), conducted by the China Social Science Survey Center at Peking University. This longitudinal study employed a multi-stage, implicit stratification, and population-scale proportional systematic probability sampling methodology. The sampling method began with multi-stage sampling conducted nationwide. In the first stage, several provinces were selected as the primary sampling units. In the second stage, cities and rural areas were drawn from these selected provinces based on population distribution as secondary sampling units. In the third stage, households and individuals were randomly selected within these secondary units as the final sample units. Implicit stratification refers to the process where no explicit groups are defined at each level of sampling; instead, the stratification occurs naturally based on population characteristics and distribution. This means that the sample’s representativeness across the country can effectively reflect the characteristics of groups from different regions and socio-economic backgrounds. The population-scale proportional systematic probability sampling method ensures that subgroups within the sample are appropriately selected based on their proportions in the overall population. Specifically, the survey adjusted the sample according to the proportions of population characteristics such as region, urban/rural areas, age, and gender, thus ensuring the broad representativeness of the final sample.

#### 3.1.2 Data cleaning and filtering

**Figure 1:**
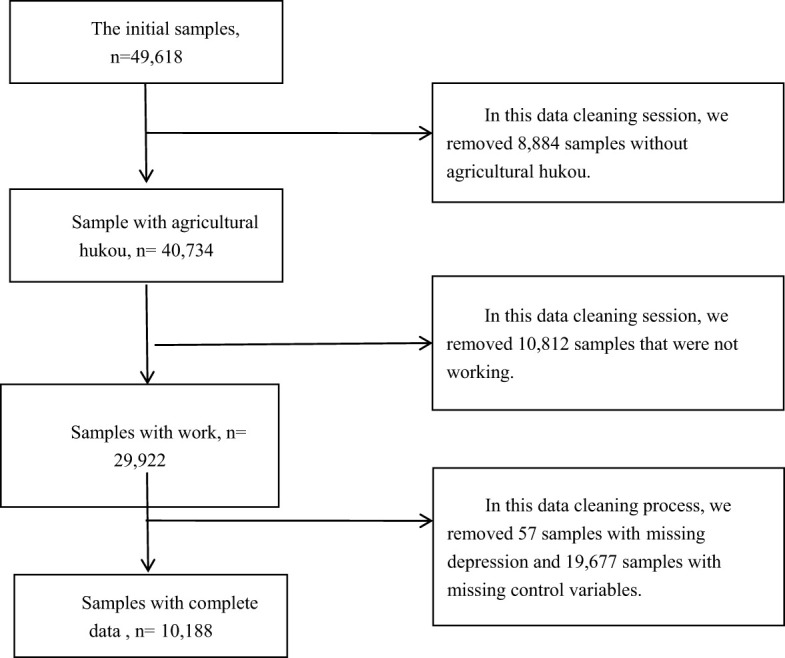
Data filtering.

The survey has been conducted since 2010, with a biennial tracking survey. Due to substantial missing values in data before 2016, this study utilizes the latest two years of survey data (2018 and 2020). Considering the applicability of the questions, this study confines its scope to the group that “engaged in work in the past week,” namely, the working labor force. Stata 17 is employed for data statistical analysis. During the data cleaning process, we first removed samples that did not meet the criteria of this study. Subsequently, samples with missing values for key variables were excluded. The specific data filtering process is shown in **Figure 1**, which clearly illustrates the steps from raw data to the final dataset. Ultimately, the effective sample size for analysis is 10,188.

### 3.2 Variables

#### 3.2.1 Dependent variable

The dependent variable of this study is the level of depression, derived from the question in the CFPS about “the frequency of various feelings or behaviors in the past week”, based on the study by Zhou et al. (32). The CFPS utilized the Center for Epidemiologic Studies Depression Scale (CES-D) to measure symptoms of depression (33), including feelings of being down, difficulty in doing anything, poor sleep quality, feelings of happiness, loneliness, enjoying life, sadness, and the belief that one cannot continue with life. Each question was rated from 1 to 4 (1=never; 2=sometimes, 1-2 days; 3=often, 3-4 days; 4=most of the time, 5-7 days), with the fourth and sixth questions requiring reverse scoring.

In 2012, CFPS had employed the CESD20, consisting of 20 questions. To facilitate a comparison of depression scores across different rounds, CFPS 2018 and 2020 randomly retained 1/5 of the respondents to use CESD20, while the remaining 4/5 used CESD8. Afterward, using percentile equalization, scores from the two sets of questions were made comparable, generating the comparable score, CESD20sc, which is the selected dependent variable for this study. Its value spectrum spans from 20 to 80, with ascending scores reflective of augmented depression intensity.

#### 3.2.2 Independent variables

The independent variables in this study encompass various types of WAH groups. Referring to Yang’s study on Chinese WAH (34), this study defined WAH based on the type of hukou. Additionally, drawing from CFPS on the “current hukou status” and work types, respondents are categorized into two main groups: WAH and WNAH. Moreover, to differentiate the work types within WAH, this study references two CFPS questions: “Is this primary job for self/family or employment by someone else?” and “Is this primary job in agriculture or non-agriculture?”. Furthermore, considering the unique dual hukou system in China (3), referencing the urban-rural classification by the National Bureau of Statistics of China, this study incorporates distinctions based on the residential location of WAH (35). Therefore, this study classifies WAH into four subgroups: Subsistence Farmers, Agricultural Laborers, RUMW, and RNAW.

It is noteworthy that this study is a comparative study among multiple groups. When comparing pairs of different WAH groups, the two sides of the comparison are respectively assigned values of 1 and 0.

#### 3.2.3 Control variables and measurements

Given the research question, control variables were selected to include age, gender, province, marital status, educational level, and political identity as objective individual characteristic variables (35); self-rated health and medical history as health variables; and assessments of popularity, trust in neighbors, life satisfaction, confidence in the future, and perceptions of income and social status as subjective individual variables (36).

**Table 1** provides the definitions of independent variables, dependent variables, control variables, and characteristics of the sample data. Concerning the dependent variable, the overall sample displays an average depression level of 32.762, signifying a relatively low level of depression. In terms of the independent variables, WAH constitutes 73.4% of the sample, forming a crucial foundation of Chinese society. Among them, Subsistence Farmers, RUMW, and RNAW constitute a considerable proportion at 10.7%, 26.7%, and 31.5%, respectively, while Agricultural Laborers constitute a smaller proportion at 4.4%. Regarding the control variables, concerning individual objective characteristic variables, the sample’s average age hovers close to 40 years old, maintaining a balanced gender distribution. Geographical distribution, however, is uneven, and a significant majority are married. The average education level of the sample corresponds to junior high school, with less than 2% of the sample being members of the Communist Party of China (CPC). Concerning health variables, the sample’s SRH level is 3.2, indicating a moderate level and 9.4% of the sample reported a history of illness in the past year. Regarding individual subjective characteristics, assessments of personal relationships, trust in neighbors, life satisfaction, and confidence in the future are generally rated at medium-high levels, while evaluations of income status and social status are rated as medium.

**Table 1:** Definitions of the variables and the descriptive statistics of the data.

Variable	Variables definition	Obs	Mean	Std. Dev.	Min	Max
depression	Each question is graded on a scale of 1 to 4 (1= never; 2= sometimes, 1-2 days; 3= often, 3-4 days; 4= most time, 5-7 days).	10188	32.762	7.246	22	72
WAH	yes=1, no =0	10188	.734	.442	0	1
Subsistence Farmers	yes=1, no=0	10188	.107	.31	0	1
Agricultural Laborers	yes=1, no=0	10188	.044	.205	0	1
RUNW	yes=1, no=0	10188	.267	.443	0	1
RNAW	yes=1, no=0	10188	.315	.464	0	1
age	age of sample	10188	39.745	11.009	16	83
gender	male=1, female=0	10188	.59	.492	0	1
province	eastern=1, non-eastern=0	10188	.355	.479	0	1
marriage	first marriage/remarriage=1, unmarried/divorced/widowed/cohabitation =0	10188	.804	.397	0	1
education	no schooling =0, primary school/private school =6, junior high school =9, Regular senior high school/vocational high school/technical school/technical secondary school =12, Junior College =15, Undergraduate =16, Master =19	10188	14.86	4.911	0	19
CPC	yes=1, no=0	10188	.016	.127	0	1
SRH	The value is proportional to the degree of SRH, assigned from 1 to 5	10188	3.239	1.082	1	5
disease history	yes=1, no=0	10188	.094	.291	0	1
popularity	The value is proportional to the degree of popularity, assigned from 1 to 5	10188	7.016	1.781	0	10
trust	The value is proportional to the degree of trust in neighbor, assigned from 1 to 5	10188	6.675	2.007	0	10
life satisfaction	The value is proportional to the degree of life satisfaction, assigned from 1 to 5	10188	3.913	.933	1	5
future confidence	The value is proportional to the degree of confidence in the future, assigned from 1 to 5	10188	4.157	.878	1	5
income status	The value is proportional to the degree of income status, assigned from 1 to 5	10188	2.847	.946	1	5
social status	The value is proportional to the degree of social status, assigned from 1 to 5	10188	2.933	.98	1	5

#### 3.2.4 Mediating variable

Based on hukou type, the internal differences among the WAH subgroups, due to variations in job type and living location, placed them in different ecosystems, leading to significant disparities, especially in terms of openness of thought. Therefore, the degree of cognitive openness was chosen as a mediating variable to explore the internal mechanisms behind the differences in depression levels among the WAH subgroups.

Since the CFPS questionnaire does not directly include questions measuring cognitive openness, this study adopted an indirect measurement approach in the selection and definition of variables, constructing an indicator of cognitive openness based on two dimensions: traditional fertility beliefs and traditional gender beliefs. Theoretically, there is a negative correlation between the degree of endorsement of traditional beliefs and cognitive openness (25). Specifically, a higher level of endorsement of traditional beliefs often indicates that an individual is more likely to maintain existing social value systems, which suggests a more closed mindset. In contrast, lower endorsement may reflect a greater acceptance of new ideas and innovations, indicating higher cognitive openness. Therefore, using reverse indicators of traditional belief endorsement to indirectly measure an individual’s cognitive openness is theoretically and methodologically valid.

In practice, this study measures the degree of endorsement of traditional beliefs using the following two questions from the CFPS questionnaire: (1) “The extent to which one agrees with the importance of having children to carry on the family lineage (i.e., the importance of lineage continuation)”; (2) “The extent to which one agrees with the statement ‘A woman’s success is less important than her marriage prospects’.” Responses to these questions range from “strongly disagree” to “strongly agree,” with scores assigned from 1 to 5, from low to high. To reflect cognitive openness, we reverse the scores for both questions, standardize them, and generate a composite index. A higher score on the final index indicates greater cognitive openness.

#### 3.2.5 Instrumental variables

As a comparative study among multiple groups, this research chose instrumental variables based on different comparison groups to address endogeneity issues. Overall, this study uses regional information to choose instrumental variables such as land ownership type (LOT), gross regional production (GRP), per capital gross regional production (PGRP), and residents’ savings deposits (RSD). LOT was derived from the CFPS “which collective land does your family get?”. Based on the type of collective land acquired, we assign a value of 1 to farmland and 0 to other types of land (including forest land, ponds, etc.). The remaining variables are from the regional economic database and regional financial database. These databases are multi-year panel databases, and considering the stability of regional economic development, data from previous years were used to supplement missing values for 2018 and 2020.

The validity of these instrumental variables is primarily reflected in two aspects: On one hand, GRP, PGRP, and RSD, as regional macroeconomic data, to some extent reflect the living standards of the WAH living in that region (37). LOT is a resource that traditional peasants rely on for survival. Thus, these instrumental variables meet the requirement of having a strong correlation with WAH. On the other hand, regional macroeconomic variables and objective factual variables are unlikely to directly influence WAH’s subjective depression levels. Therefore, these instrumental variables theoretically satisfy the requirement of exogeneity.

## 4 Results

### 4.1 Typical facts

In China, it is well known that WAH is a relatively disadvantaged position, which may have adverse effects on their mental health, especially in terms of depression levels. **Table 2** reports the depression levels of WAH and its subgroups in comparison to WNAH. Models 1-2 show that after controlling for individual objective characteristic variables, health variables, and individual subjective characteristic variables, the coefficient for depression levels is positive, indicating that the overall depression level of WAH is significantly higher than that of WNAH at the 1% level. Based on the conclusions drawn from Models 1-2, the depression levels of the four distinct subgroups were further examined. Models 3-6 demonstrate that, compared to WNAH, the depression level of Subsistence Farmers increased by 1.816 units, Agricultural Laborers by 2.926 units, RUMN by 0.958 units, and RNAW by 1.423 units, all of which are significant at the 1% level.

**Table 2:** Baseline regression results for WAH groups.

	Model 1	Model 2	Model 3	Model 4	Model 5	Model 6
	depression	depression	depression	depression	depression	depression
WAH	1.365***	1.398***				
	(0.149)	(0.141)				
Subsistence Farmers			1.816***			
			(0.247)			
Agricultural Laborers				2.926***		
				(0.327)		
RUNW					0.958***	
					(0.171)	
RNAW						1.423***
						(0.171)
age	-0.006	0.007	-0.001	-0.021*	-0.004	-0.008
	(0.007)	(0.007)	(0.012)	(0.012)	(0.009)	(0.009)
gender	-0.878***	-0.858***	-1.267***	-1.043***	-1.029***	-0.743***
	(0.141)	(0.135)	(0.214)	(0.225)	(0.172)	(0.173)
province	-1.069***	-0.982***	-0.575***	-0.816***	-0.997***	-0.941***
	(0.140)	(0.133)	(0.223)	(0.228)	(0.172)	(0.175)
marriage	-1.610***	-0.834***	-1.133***	-0.708**	-0.426*	-0.559**
	(0.201)	(0.194)	(0.301)	(0.309)	(0.237)	(0.225)
education	-0.079***	-0.090***	-0.115***	-0.103***	-0.057***	-0.069***
	(0.016)	(0.015)	(0.023)	(0.028)	(0.021)	(0.019)
CPC	-0.345	0.035	0.067	-0.623	-0.191	0.340
	(0.573)	(0.531)	(0.797)	(0.776)	(0.633)	(0.606)
SRH	-1.894***	-1.336***	-1.287***	-1.241***	-1.081***	-1.354***
	(0.071)	(0.072)	(0.108)	(0.118)	(0.090)	(0.086)
disease history	1.209***	1.170***	1.590***	1.281***	1.319***	0.924***
	(0.272)	(0.257)	(0.338)	(0.374)	(0.301)	(0.303)
popularity		-0.121***	-0.235***	-0.284***	-0.218***	-0.101**
		(0.042)	(0.065)	(0.071)	(0.054)	(0.051)
trust		-0.268***	-0.286***	-0.257***	-0.269***	-0.260***
		(0.036)	(0.055)	(0.058)	(0.046)	(0.044)
life satisfaction		-1.481***	-1.501***	-1.600***	-1.547***	-1.492***
		(0.095)	(0.139)	(0.154)	(0.118)	(0.109)
future confidence		-0.781***	-0.807***	-0.846***	-0.973***	-0.743***
		(0.099)	(0.145)	(0.156)	(0.119)	(0.114)
income status		-0.066	0.002	-0.059	-0.125	-0.093
		(0.090)	(0.137)	(0.147)	(0.115)	(0.108)
social status		-0.040	-0.116	-0.113	-0.161	-0.207*
		(0.091)	(0.138)	(0.146)	(0.114)	(0.106)
_cons	41.378***	50.509***	52.422***	53.464***	51.681***	50.832***
	(0.479)	(0.593)	(0.893)	(0.979)	(0.737)	(0.721)
F-value	120.41	131.26	67.76	59.38	93.15	87.51
Prob > F	0.0000	0.0000	0.0000	0.0000	0.0000	0.0000
N	10188	10188	3807	3161	5437	5919
r2	0.113	0.191	0.211	0.221	51.681***	50.832***

Robust standard errors are reported in parentheses; *, **, *** denote significance at the 10%, 5%, 1% levels, respectively.

These results indicate that compared to WNAH, various farmer groups perform worse in terms of depression levels, which is consistent with general understanding and serves as the factual basis for this study.

### 4.2 Baseline regression

The aforementioned facts have shown that different WAH identities, relative to WNAH, have a negative impact on individual depression levels. However, a pertinent question arises: do the depression levels differ among these four subgroups of WAH? The study embarks on comparing and analyzing the differences in depression levels among these groups.

**Table 3** presents the baseline regression results, reporting the differences in depression levels among the four WAH groups after adding a series of control variables. Firstly, Models 7-9 show the depression levels of Subsistence Farmers compared to Agricultural Laborers, RUMW, and RNAW (Subsistence Farmers=1; others=0); the results indicate that the depression level of Subsistence Farmers is lower than that of Agricultural Laborers but higher than that of RUMW, passing the 5% significance test. Compared to RNAW, the depression level of Subsistence Farmers is higher, but not significantly so. Secondly, Models 10-11 represent the depression levels of Agricultural Laborers compared to RUMW and RNAW respectively (Agricultural Laborers=1, others=0); the results show that the depression levels of Agricultural Laborers are significantly higher at the 1% level, by 1.796 and 1.431 units, respectively. Finally, Model 12 reports the regression results of depression levels between RUMW and RNAW (RUMW=1, RNAW=0), showing that the depression level of RUMW is on average 0.41 units lower at the 5% significance level.

**Table 3:** Comparison of depression levels among four WAH groups.

	Model 7	Model 8	Model 9	Model 10	Model 11	Model 12
	Agricultural Laborers depression	RUNW depression	RNAW depression	RUNW depression	RNAW depression	RNAW depression
Subsistence Farmers	-1.006**	0.540**	0.179			
	(0.415)	(0.272)	(0.263)			
Agricultural Laborers				1.796***	1.431***	
				(0.368)	(0.365)	
RUNW						-0.410**
						(0.172)
age	0.016	0.022*	0.011	0.009	-0.002	0.007
	(0.019)	(0.012)	(0.012)	(0.013)	(0.012)	(0.010)
gender	-1.145***	-1.078***	-0.684***	-0.825***	-0.428*	-0.646***
	(0.384)	(0.222)	(0.222)	(0.238)	(0.238)	(0.178)
province	-0.169	-0.880***	-0.786***	-1.153***	-1.037***	-1.122***
	(0.453)	(0.230)	(0.234)	(0.233)	(0.236)	(0.175)
marriage	-2.742***	-1.062***	-1.082***	-0.667**	-0.764**	-0.541**
	(0.691)	(0.329)	(0.312)	(0.338)	(0.321)	(0.243)
education	-0.132***	-0.088***	-0.090***	-0.075***	-0.082***	-0.057***
	(0.030)	(0.023)	(0.021)	(0.027)	(0.024)	(0.020)
CPC	-1.455	-0.062	0.877	-1.037	0.134	0.359
	(2.536)	(0.990)	(1.057)	(0.849)	(0.994)	(0.710)
SRH	-1.638***	-1.256***	-1.545***	-1.184***	-1.553***	-1.321***
	(0.187)	(0.118)	(0.110)	(0.130)	(0.121)	(0.094)
disease history	1.412**	1.523***	1.020**	1.058**	0.459	0.774**
	(0.655)	(0.436)	(0.422)	(0.491)	(0.470)	(0.363)
popularity	-0.168	-0.135**	-0.010	-0.163**	-0.006	-0.044
	(0.106)	(0.069)	(0.065)	(0.075)	(0.070)	(0.056)
trust	-0.272***	-0.292***	-0.276***	-0.258***	-0.246***	-0.261***
	(0.088)	(0.061)	(0.056)	(0.065)	(0.059)	(0.048)
life satisfaction	-1.474***	-1.450***	-1.394***	-1.541***	-1.437***	-1.463***
	(0.230)	(0.157)	(0.143)	(0.174)	(0.155)	(0.126)
future confidence	-0.622**	-0.905***	-0.597***	-0.966***	-0.626***	-0.804***
	(0.247)	(0.164)	(0.154)	(0.175)	(0.165)	(0.131)
income status	0.083	-0.053	-0.037	-0.101	-0.060	-0.123
	(0.216)	(0.157)	(0.133)	(0.169)	(0.140)	(0.120)
social status	0.421*	0.060	-0.030	0.090	-0.032	-0.102
	(0.217)	(0.159)	(0.133)	(0.171)	(0.140)	(0.121)
_cons	53.746***	51.237***	50.587***	51.627***	50.808***	50.932***
	(1.578)	(0.946)	(0.879)	(1.031)	(0.946)	(0.753)
F-value	24.06	50.81	50.62	46.21	44.44	70.63
Prob > F	0.0000	0.0000	0.0000	0.0000	0.0000	0.0000
N	1544	3820	4302	3174	3656	5932
r2	0.207	0.193	0.178	0.199	0.179	0.181

Robust standard errors are reported in parentheses; *, **, *** denote significance at the 10%, 5%, 1% levels, respectively.

Overall, the study finds that RUMW has the lowest level of depression. The difference between RNAW and Subsistence Farmers is not significant, while Agricultural Laborers have the highest level of depression. This implies that for workers with an agricultural hukou, engaging in non-agricultural work or living and working in urban areas has a certain positive effect on the depression levels of WAH.

Lastly, the regression results for the control variables are interpreted. Regarding individual objective characteristics, age, and political identity do not significantly affect an individual’s depression levels. However, male individuals, those residing in the eastern region, married individuals, and those with higher education levels have significantly lower depression levels. In terms of health characteristics, groups with a higher self-rated health level and without a history of major illnesses in the past year have significantly lower depression levels. Regarding individual subjective characteristics, Subsistence Farmers and Agricultural Laborers with a higher self-assessment of their social ties perform better only compared to RUMW, with significantly lower depression levels at the 5% level. Farmers with higher trust in neighbors, higher life satisfaction, and greater confidence in the future have lower depression levels, significant at the 1% level. The impact of income and social status perception on depression levels is not significant for most WAH groups.

### 4.3 Robustness checks

The research findings indicate significant differences in depression levels among the various subgroups of WAH. To enhance the scientific validity and credibility of the regression results, this study employs two methods for robustness checks. Firstly, the CESD8 is used instead of the CESD20sc as the indicator for measuring depression levels (results shown in **Table 4**); secondly, the sample is expanded by including the CFPS 2016 sample (results shown in **Table 5**). The robustness check results, as seen in **Tables 4**, **5**, are fundamentally consistent with the baseline regression results, showing differences in depression levels among various WAH groups.

**Table 4:** Change the calculation indicator for depression levels.

	Agricultural Laborers depression2	RUNW depression2	RNAW depression2	RUNW depression2	RNAW depression2	RNAW depression2
Subsistence Farmers	-0.509**	0.273**	0.090			
	(0.209)	(0.137)	(0.133)			
Agricultural Laborers				0.903***	0.722***	
				(0.186)	(0.184)	
RUNW						-0.205**
						(0.087)
age	0.008	0.011*	0.005	0.004	-0.001	0.003
	(0.010)	(0.006)	(0.006)	(0.006)	(0.006)	(0.005)
gender	-0.587***	-0.549***	-0.345***	-0.419***	-0.213*	-0.325***
	(0.194)	(0.112)	(0.112)	(0.120)	(0.120)	(0.090)
province	-0.102	-0.450***	-0.406***	-0.587***	-0.533***	-0.572***
	(0.229)	(0.116)	(0.118)	(0.117)	(0.120)	(0.089)
marriage	-1.379***	-0.542***	-0.543***	-0.343**	-0.383**	-0.275**
	(0.345)	(0.165)	(0.157)	(0.170)	(0.162)	(0.123)
education	-0.067***	-0.045***	-0.046***	-0.038***	-0.041***	-0.029***
	(0.015)	(0.012)	(0.011)	(0.013)	(0.012)	(0.010)
CPC	-0.837	-0.050	0.409	-0.539	0.037	0.167
	(1.264)	(0.496)	(0.533)	(0.432)	(0.505)	(0.360)
SRH	-0.824***	-0.633***	-0.782***	-0.597***	-0.786***	-0.669***
	(0.094)	(0.059)	(0.056)	(0.066)	(0.061)	(0.047)
disease history	0.707**	0.750***	0.499**	0.523**	0.225	0.374**
	(0.329)	(0.218)	(0.213)	(0.245)	(0.237)	(0.182)
popularity	-0.084	-0.069**	-0.005	-0.083**	-0.003	-0.023
	(0.053)	(0.035)	(0.033)	(0.038)	(0.035)	(0.028)
trust	-0.135***	-0.146***	-0.137***	-0.130***	-0.123***	-0.131***
	(0.044)	(0.031)	(0.028)	(0.033)	(0.030)	(0.024)
life satisfaction	-0.741***	-0.730***	-0.701***	-0.776***	-0.723***	-0.737***
	(0.115)	(0.078)	(0.072)	(0.087)	(0.078)	(0.063)
future confidence	-0.312**	-0.455***	-0.302***	-0.488***	-0.318***	-0.407***
	(0.125)	(0.082)	(0.078)	(0.088)	(0.083)	(0.066)
income status	0.042	-0.028	-0.024	-0.047	-0.032	-0.064
	(0.109)	(0.079)	(0.067)	(0.085)	(0.071)	(0.060)
social status	0.208*	0.029	-0.015	0.046	-0.014	-0.050
	(0.109)	(0.080)	(0.067)	(0.086)	(0.071)	(0.061)
_cons	23.916***	22.685***	22.358***	22.874***	22.463***	22.535***
	(0.793)	(0.476)	(0.443)	(0.517)	(0.475)	(0.378)
F-value	24.32	51.14	51.13	46.63	45.11	71.52
Prob > F	0.0000	0.0000	0.0000	0.0000	0.0000	0.0000
N	1544	3820	4302	3174	3656	5932
r2	0.206	0.193	0.177	0.199	0.178	0.181

Robust standard errors are reported in parentheses; *, **, *** denote significance at the 10%, 5%, 1% levels, respectively.

**Table 5:** Expand samples.

	Agricultural workers depression	RUNW depression	RNAW depression	RUNW depression	RNAW depression	RNAW depression
Subsistence Farmers	-0.806***	0.366**	0.076			
	(0.305)	(0.178)	(0.176)			
Agricultural Laborers				1.464***	1.073***	
				(0.277)	(0.276)	
RUNW						-0.273**
						(0.122)
age	-0.000	0.002	-0.001	-0.006	-0.010	-0.008
	(0.009)	(0.007)	(0.007)	(0.008)	(0.009)	(0.006)
gender	-1.282***	-0.884***	-0.738***	-0.638***	-0.440**	-0.476***
	(0.236)	(0.144)	(0.152)	(0.164)	(0.177)	(0.126)
province	-0.668**	-0.848***	-0.956***	-0.999***	-1.121***	-1.040***
	(0.269)	(0.147)	(0.159)	(0.158)	(0.173)	(0.123)
marriage	-2.246***	-1.244***	-1.344***	-0.900***	-1.033***	-0.810***
	(0.375)	(0.194)	(0.203)	(0.217)	(0.232)	(0.164)
education	-0.105***	-0.069***	-0.082***	-0.041**	-0.066***	-0.037***
	(0.018)	(0.014)	(0.014)	(0.018)	(0.017)	(0.014)
CPC	-0.539	-0.381	-0.236	-0.761*	-0.590	-0.322
	(0.622)	(0.400)	(0.429)	(0.457)	(0.505)	(0.362)
SRH	-1.440***	-1.298***	-1.439***	-1.145***	-1.366***	-1.265***
	(0.110)	(0.074)	(0.074)	(0.088)	(0.088)	(0.066)
disease history	1.797***	1.739***	1.550***	1.106***	0.738**	1.079***
	(0.364)	(0.263)	(0.272)	(0.324)	(0.342)	(0.253)
popularity	0.118***	0.096***	0.111***	0.081***	0.105***	0.091***
	(0.018)	(0.011)	(0.012)	(0.013)	(0.014)	(0.010)
trust	-0.377***	-0.376***	-0.331***	-0.347***	-0.283***	-0.320***
	(0.055)	(0.037)	(0.037)	(0.043)	(0.043)	(0.032)
life satisfaction	-1.351***	-1.432***	-1.246***	-1.563***	-1.282***	-1.403***
	(0.140)	(0.092)	(0.094)	(0.109)	(0.114)	(0.083)
future confidence	-0.862***	-0.934***	-0.810***	-1.000***	-0.805***	-0.900***
	(0.142)	(0.097)	(0.099)	(0.116)	(0.120)	(0.089)
income status	0.083	0.004	-0.102	0.018	-0.128	-0.110
	(0.136)	(0.096)	(0.094)	(0.111)	(0.107)	(0.083)
social status	0.195	-0.042	0.068	-0.136	0.018	-0.100
	(0.131)	(0.094)	(0.091)	(0.110)	(0.105)	(0.082)
_cons	52.604***	50.943***	50.456***	50.921***	50.078***	50.492***
	(0.894)	(0.561)	(0.559)	(0.667)	(0.660)	(0.502)
F-value	61.34	123.82	113.45	89.02	77.89	141.87
Prob > F	0.0000	0.0000	0.0000	0.0000	0.0000	0.0000
N	4258	9624	9256	7046	6678	12044
r2	0.201	0.197	0.185	0.188	0.169	0.179

Robust standard errors are reported in parentheses; *, **, *** denote significance at the 10%, 5%, 1% levels, respectively.

### 4.4 PSM test

The above research has already demonstrated the differences in depression levels among the four WAH groups, suggesting variations in their levels of depression. Considering the large objective individual differences among these four groups, the study could not entirely rule out self-selection bias issues that may influence their levels of depression. Therefore, building upon the regression analysis of the differences in their depression levels, this study will further employ the PSM method to address potential endogeneity issues.

In operational terms, first, the covariates used in the baseline regression are included. Next, the reliability of the matching results was verified, specifically whether they passed the balance test. **Tables 6**–**11** show the results of the balance test conducted pairwise among the four farmer subgroups, where the majority of the covariates have a bias smaller than 10% after matching, and most differences between the two groups are not significant, indicating that the balance tests are passed. Finally, the net effects of the differences in depression levels among the four WAH groups were estimated using three matching methods: nearest-neighbor matching, local linear regression matching, and radius matching. **Table 12** presents the average treatment effects of these three matching methods. The results consistently indicate the following: first, Subsistence Farmers have a significantly lower level of depression compared to Agricultural Laborers and higher than RUMW, with no significant result compared to RNAW. Second, Agricultural Laborers have a significantly higher level of depression compared to both RUMW and RNAW. Third, compared to RNAW, the depression level of RUMW is significantly lower. These estimated results are consistent with the baseline regression results.

**Table 6:** Balance test between subsistence farmers and agricultural laborers.

Variable	Unmatched	Mean Treated Control		Bias%	Reduct	T-value	P-value
	Matched				Bias%		
age	U	46.312	43.731	24.3		4.48	0.000
	M	46.261	46.796	-5.0	79.3	-1.21	0.226
gender	U	.63288	.58575	9.7		1.73	0.083
	M	.63119	.57431	11.7	-20.7	2.72	0.007
province	U	.19635	.31849	-28.2		-5.21	0.000
	M	.19725	.21651	-4.4	84.2	-1.11	0.267
marriage	U	.90228	.85746	13.8		2.55	0.011
	M	.90183	.91651	-4.5	67.3	-1.19	0.233
education	U	13.622	13.189	6.3		1.13	0.258
	M	13.597	13.824	-3.3	47.6	-0.80	0.426
CPC	U	.00639	.01559	-8.8		-1.73	0.083
	M	.00642	.00826	-1.8	80.1	-0.50	0.616
SRH	U	3.0913	3.2249	-11.2		-2.01	0.045
	M	3.0972	3.0413	4.7	58.1	1.10	0.270
disease history	U	.14247	.11359	8.6		1.51	0.131
	M	.13945	.15505	-4.7	46.0	-1.03	0.304
popularity	U	7.0301	7.0535	-1.2		-0.21	0.832
	M	7.0257	7.0202	0.3	76.4	0.07	0.948
trust	U	6.9132	6.7617	6.9		1.26	0.208
	M	6.9028	6.9422	-1.8	74.0	-0.42	0.672
life satisfaction	U	3.9872	4.0312	-4.5		-0.80	0.426
	M	3.9881	4.0459	-6.0	-31.5	-1.40	0.161
future confidence	U	4.221	4.2494	-3.2		-0.57	0.569
	M	4.222	4.2	2.5	22.6	0.57	0.567
income status	U	2.895	2.9555	-5.6		-1.01	0.311
	M	2.8991	2.9018	-0.3	95.4	-0.06	0.952
social status	U	3.1279	3.1648	-3.4		-0.62	0.537
	M	3.1294	3.144	-1.4	60.3	-0.32	0.748

**Table 7:** Balance test between subsistence farmers and RUMW.

Variable	Unmatched	Mean Treated Control		Bias%	Reduct	T-value	P-value
	Matched				Bias%		
age	U	46.312	38.148	78.8		21.52	0.000
	M	46.312	46.567	-2.5	96.9	-0.59	0.553
gender	U	.63288	.57431	12.0		3.33	0.001
	M	.63288	.6274	1.1	90.6	0.27	0.791
marriage	U	.90228	.80257	28.4		7.49	0.000
	M	.90228	.90594	-1.0	96.3	-0.29	0.772
education	U	13.622	15.195	-27.1		-8.26	0.000
	M	13.622	13.759	-2.4	91.3	-0.49	0.622
CPC	U	.00639	.01468	-8.1		-2.10	0.036
	M	.00639	.00365	2.7	66.9	0.91	0.365
SRH	U	3.0913	3.2906	-17.8		-5.09	0.000
	M	3.0913	3.1096	-1.6	90.8	-0.37	0.713
disease history	U	.14247	.08037	19.8		5.86	0.000
	M	.14247	.11963	7.3	63.2	1.58	0.114
popularity	U	7.0301	6.9204	5.9		1.69	0.090
	M	7.0301	7.1288	-5.3	10.1	-1.20	0.229
trust	U	6.9132	6.6283	14.3		4.04	0.000
	M	6.9132	6.989	-3.8	73.4	-0.90	0.371
life satisfaction	U	3.9872	3.8899	10.1		2.88	0.004
	M	3.9872	3.9799	0.8	92.5	0.18	0.861
future confidence	U	4.221	4.1277	10.6		2.96	0.003
	M	4.221	4.2438	-2.6	75.5	-0.60	0.548
income status	U	2.895	2.8026	9.3		2.69	0.007
	M	2.895	2.8584	3.7	60.5	0.81	0.419
social status	U	3.1279	2.8653	26.1		7.47	0.000
	M	3.1279	3.0822	4.5	82.6	1.02	0.309

**Table 8:** Balance test between subsistence farmers and RNAW.

Variable	Unmatched	Mean Treated Control		Bias%	Reduct	T-value	P-value
	Matched				Bias%		
age	U	46.312	38.044	78.4		21.64	0.000
	M	46.312	46.247	0.6	99.2	0.16	0.876	
gender	U	.63288	.63923	-1.3		-0.38	0.706	
	M	.63288	.6484	-3.2	-144.5	-0.76	0.449	
province	U	.19635	.3087	-26.1		-7.19	0.000	
	M	.19635	.17534	4.9	81.3	1.26	0.207	
marriage	U	.90228	.76988	36.3		9.62	0.000	
	M	.90228	.9105	-2.3	93.8	-0.66	0.509	
education	U	13.622	14.948	-22.1		-6.82	0.000	
	M	13.622	14.12	-8.3	62.5	-1.79	0.074	
CPC	U	.00639	.01715	-10.0		-2.58	0.010	
	M	.00639	.00274	3.4	66.0	1.27	0.205	
SRH	U	3.0913	3.3302	-20.9		-6.08	0.000	
	M	3.0913	3.1507	-5.2	75.2	-1.20	0.230	
disease history	U	.14247	.07328	22.4		6.91	0.000	
	M	.14247	.12694	5.0	77.6	1.06	0.288	
popularity	U	7.0301	7.0402	-0.5		-0.16	0.876	
	M	7.0301	7.0941	-3.4	-533.7	-0.78	0.437	
trust	U	6.9132	6.7543	7.8		2.24	0.025	
	M	6.9132	6.9452	-1.6	79.9	-0.37	0.715	
life satisfaction	U	3.9872	3.8771	11.1		3.21	0.001	
	M	3.9872	4.0018	-1.5	86.7	-0.35	0.725	
future confidence	U	4.221	4.2108	1.2		0.33	0.741	
	M	4.221	4.2027	2.1	-78.8	0.48	0.634	
income status	U	2.895	2.889	0.6		0.17	0.862	
	M	2.895	2.916	-2.1	-251.0	-0.49	0.626	
social status	U	3.1279	2.9492	17.3		5.01	0.000	
	M	3.1279	3.1808	-5.1	70.4	-1.21	0.225	

**Table 9:** Balance test between agricultural laborers and RUMW.

Variable	Unmatched	Mean Treated Control		Bias%	Reduct	T-value	P-value
	Matched				Bias%		
age	U	43.731	38.148	49.9		9.97	0.000
	M	43.692	44.174	-4.3	91.4	-0.64	0.522
gender	U	.58575	.57431	2.3		0.45	0.650
	M	.58705	.6317	-9.0	-290.4	-1.37	0.171
province	U	.31849	.42275	-21.7		-4.18	0.000
	M	.3192	.32366	-0.9	95.7	-0.14	0.886
marriage	U	.85746	.80257	14.6		2.75	0.006
	M	.85938	.87946	-5.4	63.4	-0.89	0.373
education	U	13.189	15.195	-34.2		-7.89	0.000
	M	13.219	13.25	-0.5	98.4	-0.07	0.944
CPC	U	.01559	.01468	0.7		0.15	0.882
	M	.01563	.01786	-1.8	-144.9	-0.26	0.795
SRH	U	3.2249	3.2906	-5.8		-1.20	0.231
	M	3.2299	3.1853	3.9	32.0	0.57	0.571
disease history	U	.11359	.08037	11.2		2.34	0.019
	M	.11161	.10938	0.8	93.3	0.11	0.915
popularity	U	7.0535	6.9204	7.1		1.46	0.145
	M	7.0469	7.0603	-0.7	89.9	-0.10	0.918
trust	U	6.7617	6.6283	6.2		1.31	0.191
	M	6.7656	6.8237	-2.7	56.5	-0.41	0.685
life satisfaction	U	4.0312	3.8899	15.2		3.01	0.003
	M	4.029	3.9554	7.9	47.9	1.17	0.243
future confidence	U	4.2494	4.1277	13.6		2.70	0.007
	M	4.2478	4.2455	0.2	98.2	0.04	0.970
income status	U	2.9555	2.8026	15.1		3.16	0.002
	M	2.9509	2.8951	5.5	63.5	0.80	0.424
social status	U	3.1648	2.8653	29.3		6.06	0.000
	M	3.1607	3.1049	5.5	81.4	0.80	0.424

**Table 10:** Balance test between agricultural laborers and RNAW.

Variable	Unmatched	Mean Treated Control		Bias%	Reduct	T-value	P-value
	Matched				Bias%		
age	U	43.731	38.044	50.1		9.99	0.000
	M	43.731	43.45	2.5	95.1	0.37	0.712
gender	U	.58575	.63923	-11.0		-2.20	0.028
	M	.58575	.57016	3.2	70.8	0.47	0.637
province	U	.31849	.3087	2.1		0.42	0.675
	M	.31849	.2784	8.6	-309.7	1.31	0.190
marriage	U	.85746	.76988	22.6		4.21	0.000
	M	.85746	.88864	-8.1	64.4	-1.40	0.161
education	U	13.189	14.948	-29.1		-6.55	0.000
	M	13.189	13.071	1.9	93.3	0.26	0.797
CPC	U	.01559	.01715	-1.2		-0.24	0.811
	M	.01559	.00668	7.0	-471.1	1.27	0.204
SRH	U	3.2249	3.3302	-9.1		-1.87	0.061
	M	3.2249	3.2472	-1.9	78.8	-0.28	0.778
disease history	U	.11359	.07328	13.9		2.98	0.003
	M	.11359	.12027	-2.3	83.4	-0.31	0.756
popularity	U	7.0535	7.0402	0.7		0.14	0.887
	M	7.0535	6.9666	4.5	-556.7	0.64	0.522
trust	U	6.7617	6.7543	0.3		0.07	0.943
	M	6.7617	6.7439	0.8	-140.6	0.12	0.907
life satisfaction	U	4.0312	3.8771	16.1		3.16	0.002
	M	4.0312	4.0757	-4.7	71.1	-0.69	0.488
future confidence	U	4.2494	4.2108	4.3		0.87	0.387
	M	4.2494	4.2695	-2.2	48.1	-0.33	0.745
income status	U	2.9555	2.889	6.4		1.35	0.177
	M	2.9555	2.9065	4.8	26.3	0.68	0.495
social status	U	3.1648	2.9492	20.6		4.21	0.000
	M	3.1648	3.0624	9.8	52.5	1.39	0.165

**Table 11:** Balance test between RUMW and RNAW.

Variable	Unmatched	Mean Treated Control		Bias%	Reduct	T-value	P-value
	Matched				Bias%		
age	U	38.148	38.044	0.9		0.36	0.719
	M	38.151	38.255	-0.9	1.0	-0.34	0.731
gender	U	.57431	.63923	-13.3		-5.12	0.000
	M	.57494	.57605	-0.2	98.3	-0.08	0.934
education	U	15.195	14.948	5.1		1.95	0.052
	M	15.191	15.252	-1.3	75.1	-0.47	0.635
marriage	U	.80257	.76988	8.0		3.06	0.002
	M	.80272	.79611	1.6	79.8	0.61	0.542
CPC	U	.01468	.01715	-2.0		-0.76	0.450
	M	.0147	.00771	5.6	-182.5	2.45	0.014
SRH	U	3.2906	3.3302	-3.7		-1.40	0.160
	M	3.2917	3.2888	0.3	92.6	0.10	0.919
disease history	U	.08037	.07328	2.7		1.02	0.306
	M	.08046	.07899	0.6	79.3	0.20	0.841
popularity	U	6.9204	7.0402	-6.7		-2.57	0.010
	M	6.9236	6.9225	0.1	99.1	0.02	0.982
trust	U	6.6283	6.7543	-6.4		-2.44	0.015
	M	6.6352	6.6447	-0.5	92.4	-0.18	0.857
life satisfaction	U	3.8899	3.8771	1.3		0.52	0.605
	M	3.8894	3.8902	-0.1	94.2	-0.03	0.977
future confidence	U	4.1277	4.2108	-9.4		-3.61	0.000
	M	4.1312	4.1076	2.7	71.7	0.97	0.333
Income status	U	2.8026	2.889	-9.2		-3.52	0.000
	M	2.8046	2.8553	-5.4	41.3	-1.99	0.046
social status	U	2.8653	2.9492	-8.6		-3.28	0.001
	M	2.8674	2.8619	0.6	93.4	0.21	0.835

**Table 12:** Average treatment effects for the treatment group.

Group	Matching method	Treatment group	Control group	ATT	Bootstrap standard error	T-value
Subsistence Farmers & Agricultural Laborers	nearest-neighbor matching	33.583	34.867	-1.283**	.618	-2.23
	local linear regression matching	33.583	34.840	-1.256***	.468	-2.18
	radius matching	33.576	34.736	-1.160**	.467	-2.38
Subsistence Farmers & RUMW	nearest-neighbor matching	33.585	32.589	.996**	.465	2.54
	local linear regression matching	33.585	32.602	.983***	.295	2.50
	radius matching	33.585	32.590	.995***	.306	3.29
Subsistence Farmers & RUMW	nearest-neighbor matching	33.585	33.936	.648	.466	1.69
	local linear regression matching	33.585	33.237	.348	.291	0.91
	radius matching	33.601	33.193	.407	.291	1.37
Agricultural Laborers & RUMW	nearest-neighbor matching	34.422	32.545	1.877***	.623	3.49
	local linear regression matching	34.422	32.667	1.755***	.386	3.27
	radius matching	34.424	32.580	1.844***	.403	4.45
Agricultural Laborers & RNAW	nearest-neighbor matching	34.465	33.187	1.278**	.622	2.30
	local linear regression matching	34.465	33.031	1.435***	.379	2.59
	radius matching	34.480	32.866	1.614***	.375	3.94
RUMW & RNAW	nearest-neighbor matching	32.658	33.324	-.666**	.282	-2.60
	local linear regression matching	32.658	33.269	-.612***	.192	-2.39
	radius matching	32.658	33.134	-.476***	.183	-2.51

Robust standard errors are reported in parentheses; **, *** denote significance at the 5%, 1% levels, respectively.

### 4.5 Instrumental variable method

Considering the potential issue of reverse causality in the research, the study attempts to address it using the instrumental variable (IV) approach. Based on the CFPS database used in the aforementioned research, we introduced and merged external databases, adding individual-level micro variables and region-level macro variables, with the merged effective sample size being 6,862. It’s important to note that due to the integration of external databases, the sample size has changed, and the preliminary estimates using OLS do not entirely align with the baseline regression results.

**Table 13** reports the two-stage least square (2SLS) estimation results of the instrumental variables for different types of WAH groups. Column (1) presents the baseline regression results. Column (2) shows the first-stage regression results of the instrumental variables along with the F-statistics values, all of which are greater than 10, indicating that the selected instrumental variables are not “weak instruments”; column (3) displays the second-stage regression results using the instrumental variables, revealing the differences in depression levels among different groups of WAH after estimating with instrumental variables. Specifically, Subsistence Farmers have a significantly lower level of depression compared to Agricultural Laborers and higher than RUMW, with the baseline regression and second-stage results consistent. Additionally, for comparisons between Subsistence Farmers and RNAW, RUMW, and RNAW, the baseline regression results and second-stage results are not consistent. This indicates the presence of endogeneity issues when comparing these groups’ depression levels, leading to biased and inconsistent OLS estimates (38). According to the study by Zahid et al. (39), controlling for endogeneity with instrumental variables to obtain asymptotically unbiased results should be the standard. Thus, after controlling for endogeneity, the level of depression in Subsistence Farmers is higher than in RNAW, and the level of depression in RUMW is lower than that of RNAW.

Combining the above results and analyzing the group differences among the four WAH groups, it is evident that there are significant differences in depression levels among the four groups, which differ in terms of work type and place of residence. This confirms Hypothesis 1.

Finally, by examining the absolute values of the coefficients in columns (1) and (3) of **Table 13**, it is observed that in the second stage of regression, the estimated coefficients for comparisons among different WAH groups are significantly larger than those in the baseline regression. This indicates the presence of potential endogeneity issues in the study of depression level differences among different WAH groups, resulting in an underestimation of the varying impact of different WAH identities on depression levels.

**Table 13:** Treatment effect using instrumental variables - 2SLS method.

Group		(1)	(2)	(3)
		Baseline Regression	the first-stage regression	the second-stage regression
Subsistence Farmers & Agricultural Laborers	Subsistence Farmers	-1.207***		-3.732*
		(0.447)		(2.251)
	LOT		0.356***	
			(0.048)	
	Control variable	yes	yes	yes
	F-statistics		55.18	
Subsistence Farmers & RUMW	Subsistence Farmers	0.634**		5.470***
		(0.304)		(1.456)
	GRP		-1.03e***	
			(3.02e)	
	RSD		-1.80e***	
			(5.50e)	
	Control variable	yes	yes	yes
	F-statistics		77.32	
Subsistence Farmers & RNAW	Subsistence Farmers	0.245		5.893***
		(0.287)		(1.945)
	GDP		7.06e***	
			(1.97e)	
	RSD		-4.19e***	
			(5.30e)	
	Control variable	yes	yes	yes
	F-statistics		57.77	
Agricultural Laborers & RUMW	Agricultural Laborers	1.968***		5.270**
		(0.400)		(2.582)
	GDP		-8.37e**	
			(3.80e)	
	Control variable	yes	yes	yes
	F-statistics		36.56	
Agricultural Laborers & RNAW	Agricultural Laborers	1.715***		22.663**
		(0.408)		(9.267)
	GDP		-3.42e***	
			(8.09e)	
	Control variable	yes	yes	yes
	F-statistics		17.85	
RUMW & RNAW	RUMW	-0.264		-10.213***
		(0.205)		(3.525)
	GDP		5.95e***	
			(1.34e)	
	Control variable	yes	yes	yes
	F-statistics		19.72	

Robust standard errors are reported in parentheses; *, **, *** denote significance at the 10%, 5%, 1% levels, respectively.

### 4.6 Mechanism analysis of depression level differences among four WAH groups

As previously analyzed, there is a considerable difference in depression levels among the different WAH groups. This study will further analyze the mechanisms influencing these differences. Given the differences in work types and living locations among the various WAH groups, which may be reflected at the cognitive level, this research selects the degree of cognitive openness as the mechanism for analysis. The results are shown in **Tables 14** and **15**. **Table 15** reports the results of using the OLS model to estimate the impact of the core explanatory variables on the mediator variable, while **Table 15** reports the regression results after including both explanatory and mediator variables. **Table 15** indicates that for each WAH group, there is a significant negative correlation between the degree of cognitive openness and their depression levels; that is, the higher the degree of cognitive openness, the lower the depression level.

Combining **Tables 14** and **15** reveals that first, Columns 1 in **Tables 14** and **15** indicate that the degree of cognitive openness cannot serve as a mechanism for analyzing the difference in depression levels between Subsistence Farmers and Agricultural Laborers. Second, Columns 2 and 3 in **Table 14** show that, compared to RUMW, Subsistence Farmers and Agricultural Laborers exhibit lower levels of cognitive openness, thus having higher levels of depression (as shown in Columns 2 and 3 of **Table 15**). Third, according to the analysis, compared to RNAW, engaging in agricultural work has a negative impact on the cognitive openness level (as shown in Column 4 of **Table 14**) and depression level (as shown in Column 4 of **Table 15**) of Agricultural Laborers; however, for RUMW, living in urban areas leads to better performance in terms of cognitive openness (as shown in Column 5 of **Table 14**) and depression levels (as shown in Column 5 of **Table 15**). These results validate Hypothesis 2.

**Table 14:** Estimation results of explanatory variables on mediating variable (cognitive openness).

Group	Explanatory Variables	(1)	(2)	(3)	(4)	(4)
		Cognitive Openness	Cognitive Openness	Cognitive Openness	Cognitive Openness	Cognitive Openness
Subsistence Farmers & Agricultural Laborers	Subsistence Farmers	-0.057				
		(0.083)				
Subsistence Farmers & RUMW	Subsistence Farmers		-0.390***			
			(0.058)			
Agricultural Laborers & RUMW	Agricultural Laborers			-0.405***		
				(0.080)		
Agricultural Laborers & RNAW	Agricultural Laborers				-0.287***	
					(0.077)	
RUMW & RNAW	RUMW					0.187***
						(0.042)
_cons		1.791***	3.240***	3.420***	2.813***	3.147***
		(0.315)	(0.204)	(0.232)	(0.220)	(0.177)
F-value		12.90	63.91	50.83	45.49	76.62
Prob > F		0.0000	0.0000	0.0000	0.0000	0.0000
N		1268	3082	2572	2968	4782
r2		0.130	0.226	0.216	0.179	0.187

Robust standard errors are reported in parentheses; *** denote significance at the 1% levels.

**Table 15:** Estimation results after incorporating explanatory and mediating variables (cognitive openness).

Group		(1) depression	(2) depression	(3) depression	(4) depression	(5) depression
Subsistence Farmers & Agricultural Laborers	Subsistence Farmers	-1.167**				
		(0.455)				
	Cognitive Openness	-0.564***				
		(0.160)				
Subsistence Farmers & RUMW	Subsistence Farmers		0.347			
			(0.305)			
	Cognitive Openness		-0.491***			
			(0.090)			
Agricultural Laborers & RUMW	Agricultural Laborers			1.746***		
				(0.404)		
	Cognitive Openness			-0.533***		
				(0.095)		
Agricultural Laborers & RNAW	Agricultural Laborers				1.337***	
					(0.396)	
	Cognitive Openness				-0.610***	
					(0.090)	
RUMW & RNAW	RUMW					-0.401**
						(0.191)
	Cognitive Openness					-0.539***
						(0.069)
_cons		55.494***	53.395***	53.251***	53.282***	53.424***
		(1.708)	(1.077)	(1.168)	(1.057)	(0.855)
F-value		22.68	42.76	38.89	39.50	59.35
Prob > F		0.0000	0.0000	0.0000	0.0000	0.0000
N		1268	3082	2572	2968	4782
r2		0.219	0.206	0.211	0.198	0.197

Robust standard errors are reported in parentheses; **, *** denote significance at the 5%, 1% levels, respectively.

## 5 Discussion

### 5.1 Significant differences exist in the depression levels among WAH

This research provides compelling empirical evidence that in China, compared to WNAH, the four distinct subgroups of WAH display higher levels of depression, aligning with findings from Li (40) and others. Building on this, by classifying farmer groups based on job type and place of residence, it’s discovered that different types of WAH exhibit varying levels of depression due to their positions within distinct ecosystems.

Firstly, significant mental health disparities exist between Subsistence Farmers and Agricultural Laborers, both engaged in agricultural work and living in rural areas. Specifically, Subsistence Farmers, possessing land resources, not only have a stable foundation for production and livelihood (39) but also enjoy greater autonomy in their work and leisure (41). From the perspective of Ecosystem Mental Health Theory at the individual level, Subsistence Farmers compared to Agricultural Laborers have more autonomy and control, which are crucial protective factors for mental health. This autonomy might be a key factor in why Subsistence Farmers have lower levels of depression compared to Agricultural Laborers. In contrast, Agricultural Laborers lack the stability brought about by land ownership and employment status, often facing higher work pressures and lower life satisfaction (42), contributing to their higher depression levels.

Secondly, the depression levels of Subsistence Farmers and Agricultural Laborers engaged in agricultural work are higher than RUMW and RNAW engaged in non-agricultural jobs. Numerous studies have already established that mental health disorders are more prevalent among agricultural communities (43). This is not just because agricultural work is susceptible to climate change and natural disasters (44), but also because the income from agricultural work is significantly lower than that from non-agricultural jobs (45). It’s evident that the adverse conditions in the work environment and income for agricultural work create greater life, work, and psychological stress for Subsistence Farmers and Agricultural Laborers, leading to higher depression levels. These findings reinforce the application of the Ecosystem Mental Health Theory in explaining the state of individual mental health, especially when considering the environmental and economic pressures faced by Agricultural workers.

Lastly, the three categories of rural farmers exhibit higher depression levels compared to urban migrant workers. Considering the environmental aspect of Ecosystem Mental Health Theory, characteristics of rural environments—such as remote locations, limited natural resources, and insufficient infrastructure—directly affect residents’ living and economic conditions. These environmental factors restrict the economic development potential of rural areas, resulting in generally lower incomes for Subsistence Farmers, Agricultural Laborers, and RNAW compared to RNUM. The income disparity not only impacts their quality of life but also increases stress and economic insecurity, which are risk factors for depression (46). Moreover, the lack of mental health professionals in rural areas hinders these three categories of rural farmers from seeking help for mental health issues.

Through the above analysis, it is evident that there are significant differences in depression levels among different subgroups of WAH due to environmental factors such as work type and living location. However, the introduction of control variables further enriches the explanation of these differences. According to the regression analysis results, in terms of individual objective characteristics, age, and political identity do not have a significant impact on an individual’s depression level. This may suggest that the direct association between depression levels and these objective characteristics is weak. This result is consistent with the studies by Zülke et al. (47) and Kim et al. (48). Men, residents in the eastern regions, married individuals, and those with higher education levels exhibit significantly lower depression levels. This reflects the impact of social support and social resources on mental health. For instance, economically developed areas in eastern China have better medical resources, higher education levels, and more comprehensive social support systems, which provide residents with a higher quality of life, better mental health support, and more opportunities to alleviate stress, thereby reducing depression levels (49). Additionally, individuals with higher education levels typically possess stronger coping skills, enabling them to adopt effective strategies to deal with stress, recognize problems, and find solutions (50). Moreover, highly educated individuals often have better economic conditions and social resources, which may reduce the risk of depression caused by financial stress (49). Regarding health characteristics, individuals with better self-rated health and no major illnesses in the past year exhibit significantly lower depression levels. This emphasizes the close connection between physical health and mental health, with good physical health possibly serving as an important protective factor for mental health (51). In terms of individual subjective characteristics, Subsistence Farmers and Agricultural Laborers, who have higher self-ratings of social relationships, show significantly lower depression levels compared to rural-to-urban migrant workers. This may indicate that positive interpersonal relationships and social support play an important role in alleviating depressive symptoms (52). WAH who have higher levels of trust in their neighbors, greater life satisfaction, and more confidence in the future exhibit lower depression levels, and this is statistically significant at the 1% level. These findings highlight the protective role of positive psychological states and social trust in mental health (53). For some WAH subgroups, depression levels are not significantly influenced by income and social status evaluation. This may imply that, for these groups, economic conditions and social status are not the primary determinants of depression. This is also supported by the studies of Bjornesta et al. (54) and Joo and Roh (55).

### 5.2 The mediating role of cognitive openness

Given the classification of WAH is based on job type and residence location, the differences in depression levels among these subgroups are closely related to these two factors. Thus, in examining the role of cognitive openness in the depression levels among WAH, job type, and residence location are key explanatory and analytical factors. In other words, the differences between agricultural and non-agricultural work, rural and urban settings, or individual and environmental differences, lead to significant distinctions in cognitive openness and depression levels among the WAH group. Firstly, job type significantly affects cognitive openness and depression levels. Agriculture, often tied to the land, binds the farmer subgroups engaged in farming to their fields, subjecting them to traditional views and societal opinions over the long term. This not only renders their thinking more conservative and traditional (44) but also prone to depression and other psychological issues (28). Moreover, influenced by traditional Chinese thoughts, they view mental illness as a shame, tending to hide their emotions and feelings (28), hindering their ability to seek help to reduce depression levels.

Secondly, the place of residence also significantly impacts cognitive openness and depression levels. On one hand, rural areas, due to their closed environment, still harbor strong adverse traditional thoughts. For instance, traditional discrimination in childbirth constitutes a direct and indirect source of psychological stress (56). On the other hand, The vast rural areas of China not only lack mental health resources (57) but also have a general lack of awareness about mental illness among their inhabitants (26). This means that residents in rural areas, while susceptible to psychological stress brought by traditional ideologies, also struggle to access professional and timely treatment, further exacerbating the severity of psychological issues (44). Meanwhile, in urban areas, an open living environment provides residents with advantages in public services and emotional support. They are less affected by adverse traditional thoughts, such as the stigmatization of mental illness, and have better access to medical treatment, thus having relatively lower levels of depression (58).

Finally, after analyzing the differences in job type and residence location, the discussion will further address the differences among WAH. Firstly, for Subsistence Farmers and Agricultural Laborers, their high similarity in job type and residence location means minor differences in cognitive openness, thus a lesser impact on depression levels. Secondly, compared to RUMW, Subsistence Farmers, Agricultural Laborers, and RNAW residing in rural areas exhibit higher levels of depression due to significant disadvantages in job type and residence location. Thirdly, for Agricultural Laborers and RNAW who also live in rural areas, the difference in job type results in higher depression levels for Agricultural Laborers engaged in agricultural work.

## 6 Conclusions

As an important social stratum in China, the physical and mental health status of WAH has always been a focus of researchers. Building on existing studies, this research, guided by the framework of the Ecosystem Mental Health Theory, primarily explores how individual psychological health statuses are influenced by multilevel factors within their ecosystems, focusing on individual and environmental levels. Moreover, the study utilizes a mixed cross-sectional dataset from 2018 and 2020 from the CFPS to examine workers with a primary job in the last year, employing the OLS model to test the impact of diverse WAH identities on depression levels and the differences among them. The empirical results indicate that (1) The regression analysis for the complete sample demonstrates significantly higher depression levels for the four WAH groups when compared to the WNAH. (2) There are differences in depression levels among the four WAH groups: RUMW have the lowest level of depression, Subsistence Farmers have higher levels of depression than RUMW and RNAW, and Agricultural Laborers have the highest level of depression. (3)Further mediation analysis reveals differences in cognitive openness among the four WAH groups, stemming from differing work types and residential locations, subsequently resulting in varying levels of depression. By analyzing the depression levels among different WAH groups and the pathways therein, this paper elucidates the mediating mechanism of cognitive openness on the depression levels of the WAH group, providing a theoretical foundation for the government to formulate differentiated policies for different types of WAH to enhance their social welfare and thus reduce their levels of depression.

The contributions and significance of this study are reflected in both theoretical and practical aspects. From a theoretical perspective, firstly, the study verifies the health issues of China’s farmer population as the research subject, expanding the application of the ecosystem health theory. Previous research has mainly focused on the mental health of urban populations, while this study applies the theory to the farmer population, specifically examining the impact of social culture on mental health, thereby enriching the application of existing theories in the context of farmers. Secondly, through an in-depth analysis of the differences in depression levels across different subgroups of workers with agricultural hukou (WAH), this study reveals the mediating role of individual differences in cognitive openness on depression, further enriching the theoretical framework of mental health research among farmers. This perspective provides a new cognitive and cultural influence viewpoint, in addition to traditional socio-economic factors and environmental stress, promoting a multidimensional understanding of the mental health of the farmer population. Finally, unlike previous studies that focused on the farmer population as a whole, this research deepens the content of mental health studies among farmers by analyzing the depression level differences among different subgroups of WAH. From a practical perspective, the findings of this study provide important references for public sectors to offer personalized support to different subgroups of WAH and provide a theoretical basis for optimizing and implementing relevant policies.

To reduce the level of depression among WAH and other vulnerable populations, this study proposes the following recommendations: First, compared to WNAH, WAH have higher levels of depression, stemming from the occupational characteristics of farmers who have long been in a disadvantageous position in life and work. Therefore, the government should genuinely safeguard the welfare of WAH in their life and work, not only eliminating the long-standing social prejudice against farmers but also ensuring and increasing their income. Second, compared to RUMW and RANW, Subsistence Farmers and Agricultural Laborers have higher levels of depression because they are more susceptible to harm from environmental changes when engaged in agricultural work. Thus, the government should provide certain protections to Subsistence Farmers and Agricultural laborer groups in the event of natural disasters. Third, compared to the three WAH groups in rural areas, RUMW, living in towns, have lower levels of depression. Therefore, the government should ensure that WAH in rural areas have access to higher levels of medical, educational, and public services, while further advancing the urbanization process. Fourth, the negative impact of WAH identities on individual depression levels mainly operates through a lower degree of cognitive openness. Hence, society needs to break the mental shackles of farmers in rural areas engaged in agricultural work by enhancing their cognitive openness through education and propaganda, thereby reducing their levels of depression.

The main limitations of this study are as follows: (1) The study did not use panel data, making it difficult to analyze the trends and causal relationships of depression levels among various farmer subgroups over time. Expanding the time window and conducting longitudinal research is the direction of effort for the next phase. (2) Although the study employed the two-stage least squares method to estimate the differences in depression levels among WAH groups to reduce estimation bias, it could not completely resolve all endogeneity issues. Future research will consider using other methods for more credible endogeneity treatment. (3) While the study considered cognitive openness as a mediator to analyze differences in depression levels among WAH, other potential mediators were not fully considered. The next phase will conduct a more comprehensive mediation analysis.

## Data Availability

Publicly available datasets were analyzed in this study. This data can be available online at CFPS [http://www.isss.pku.edu.cn/cfps/].
