# Supplementary material for: Where you live, what you do: depression differences among diverse Chinese nongmin through cognitive openness
Source: Front Psychiatry. 2025 Jan 31;16:1433949. doi: 10.3389/fpsyt.2025.1433949 (PMC11825455; doi:10.3389/fpsyt.2025.1433949)
Supplement: Supplementary file 1 [file Table1.docx]

**Supplementary files**

To enhance the scientific validity and credibility of the regression results, this study employs other methods for robustness checks. Regression analysis was conducted using the Logit model (results shown in Table 16).

Table 16 Use the Logit Model

|  | Agricultural Laborers  depression | RUNW  depression | RNAW  depression | RUNW  depression | RNAW  depression | RNAW  depression |
| --- | --- | --- | --- | --- | --- | --- |
| Subsistence farmers | -0.221* | 0.211** | 0.172** |  |  |  |
|  | (0.130) | (0.093) | (0.087) |  |  |  |
| Agricultural Laborers |  |  |  | 0.467*** | 0.402*** |  |
|  |  |  |  | (0.123) | (0.118) |  |
| RUNW |  |  |  |  |  | -0.116* |
|  |  |  |  |  |  | (0.064) |
| age | 0.010* | 0.009** | 0.004 | 0.008* | 0.004 | 0.004 |
|  | (0.006) | (0.004) | (0.004) | (0.004) | (0.004) | (0.003) |
| gender | -0.363*** | -0.330*** | -0.281*** | -0.215** | -0.186** | -0.209*** |
|  | (0.119) | (0.080) | (0.077) | (0.088) | (0.084) | (0.065) |
| province | -0.148 | -0.364*** | -0.152* | -0.431*** | -0.190** |  |
|  | (0.141) | (0.085) | (0.083) | (0.090) | (0.087) |  |
| marriage |  | -0.403*** | -0.338*** | -0.364*** | -0.293*** | -0.256*** |
|  |  | (0.111) | (0.101) | (0.118) | (0.106) | (0.085) |
| education |  | -0.023*** | -0.025*** | -0.021** | -0.025*** | -0.021*** |
|  |  | (0.007) | (0.007) | (0.009) | (0.008) | (0.007) |
| CPC | -0.295 | -0.101 | 0.355 | -0.218 | 0.297 | 0.233 |
|  | (0.621) | (0.351) | (0.321) | (0.361) | (0.322) | (0.255) |
| SRH | -0.367*** | -0.335*** | -0.428*** | -0.311*** | -0.435*** | -0.388*** |
|  | (0.058) | (0.041) | (0.038) | (0.047) | (0.042) | (0.034) |
| disease  history | 0.484*** | 0.428*** | 0.286** | 0.237 | 0.078 | 0.163 |
|  | (0.169) | (0.124) | (0.120) | (0.146) | (0.140) | (0.111) |
| popularity | -0.029 | -0.028 | -0.014 | -0.029 | -0.013 | -0.017 |
|  | (0.033) | (0.025) | (0.022) | (0.028) | (0.025) | (0.020) |
| trust | -0.085*** | -0.081*** | -0.077*** | -0.070*** | -0.067*** | -0.073*** |
|  | (0.028) | (0.022) | (0.019) | (0.024) | (0.021) | (0.017) |
| life  satisfaction | -0.302*** | -0.362*** | -0.336*** | -0.445*** | -0.381*** | -0.410*** |
|  | (0.069) | (0.050) | (0.044) | (0.058) | (0.049) | (0.041) |
| future  confidence | -0.158** | -0.222*** | -0.139*** | -0.248*** | -0.159*** | -0.185*** |
|  | (0.074) | (0.051) | (0.047) | (0.056) | (0.050) | (0.041) |
| income  status | 0.036 | -0.027 | 0.008 | -0.042 | -0.002 | -0.031 |
|  | (0.069) | (0.055) | (0.048) | (0.061) | (0.052) | (0.045) |
| social status | 0.105 | 0.043 | -0.017 | 0.025 | -0.045 | -0.036 |
|  | (0.069) | (0.056) | (0.046) | (0.061) | (0.048) | (0.043) |
| _cons | 2.448*** | 3.527*** | 3.476*** | 3.819*** | 3.745*** | 3.797*** |
|  | (0.459) | (0.326) | (0.304) | (0.360) | (0.334) | (0.272) |
| N | 1544 | 3820 | 4302 | 3174 | 3656 | 5932 |
| r2 | 0.094 | 0.109 | 0.104 | 0.118 | 0.109 | 0.105 |

Note: Robust standard errors are reported in parentheses; *, **, *** denote significance at the 10%, 5%, 1% levels, respectively.
